# Effects of the Size and Loading of Chrome-Tanned Leather Shavings on the Properties of Styrene–Butadiene Rubber Compounds

**DOI:** 10.3390/polym17091136

**Published:** 2025-04-22

**Authors:** L. R. Melo de Lima, S. Neves, S. Pinho, C. Coelho, M. F. Almeida, M. A. Lopes, C. Fonseca

**Affiliations:** 1 FEUP, Department of Mechanical Engineering, University of Porto, Rua Dr. Roberto Frias, s/n, 4200-465 Porto, Portugal; 2 LEPABE, FEUP, Department of Mechanical Engineering, University of Porto, Rua Dr. Roberto Frias, s/n, 4200-465 Porto, Portugal; 3 Atlanta—Componentes para o Calçado, Lda., AP. 57, Marco de Simães, 4615-909 Lixa, Portugal; 4 LAQV-REQUIMTE, FEUP, Department of Mechanical Engineering, University of Porto, Rua Dr. Roberto Frias, s/n, 4200-465 Porto, Portugal; 5 LAETA/INEGI, FEUP, Department of Mechanical Engineering, University of Porto, Rua Dr. Roberto Frias, s/n, 4200-465 Porto, Portugal

**Keywords:** leather, waste, leather fiber, recycling, composite, SBR

## Abstract

Among the proposed leather recycling options, the incorporation of leather waste in rubber has been the subject of multiple studies, where the effects of leather content on the mechanical and rheological properties of the composites are usually studied. However, the effects of leather size have never been addressed in a systematic way. The reasons to study this parameter are twofold: it affects the physicochemical properties and processing conditions of the composites, and leather grinding is a costly and time-consuming process. In this work, leather waste (LW) with particle sizes ranging from ≤0.5 mm to ≤3 mm was incorporated in styrene–butadiene rubber (SBR) in contents of up to 50 phr. It was concluded that the composites with finer leather sizes exhibited a more uniform particles dispersion, and tensile strength was not significantly affected by the presence of LW, especially for the finer granulometries. However, there is a remarkable increase in the stiffness with the increase in leather content, particularly with the finer particles. The abrasion increased with the incorporation of leather across all particle sizes, especially for the ≤0.5 mm leather particles. The thermal stability of the composites was not affected by either the particle size or the amount of LW, except for high contents.

## 1. Introduction

Leather is a flexible, breathable, pleasant to the touch, and durable material, which explains its preferred use in a wide range of applications, including footwear, clothing, bags, car upholstery, and sports. The importance of the leather industry to the economy is evidenced by 1.7 billion m^2^ of leather being produced annually, with an estimated market price of 34 billion dollars [1]. The durability and mechanical properties of leather, namely, its immunity to biodegradation, are achieved through the tanning process, in which the cross-linking of the collagen chains is promoted, usually with chromium [2,3,4]; however, chromium is an environmentally dangerous metal, especially when it oxidizes to Cr(VI), a carcinogenic and water-soluble form of chromium [4,5]. Therefore, the disposal of leather products and scraps in landfills, an often-adopted solution, raises serious problems related to soil and groundwater contamination by leather leachates [2,6].

Alternative solutions to this problem include thermal recycling processes such as incineration and pyrolysis. Incineration produces energy that can be used in leather processing, but it is also responsible for greenhouse emissions and releasing of toxic gases (aromatic organic compounds) [2]. Pyrolysis, in turn, is noted to allow for gas and oil production with commercial value and activated carbon, a valuable material for catalysis applications, drinking water treatment, and wastewater treatment. However, the high initial investment costs, energy demands, environmental risks, and strict regulatory challenges may limit its application.

Leather dechroming, which consists of the separation of chromium from leather, is an alternative leather valorisation process [7,8]. The recovered protein hydrolysate finds many applications, ranging from leather manufacturing, agriculture, and the pharmaceutical industry to animal feed or adhesives. The main disadvantages of this approach lie in the harsh acid or alkaline conditions that are needed and the generation of liquid effluents containing chromium. Leather scraps also find applications as adsorbents for dye or oil, allowing their removal from effluents, for example, in tanneries [5,9,10].

The incorporation of leather waste (LW) in polymer matrices for reinforcing purposes has also been widely studied in the literature. If an expressive amount of LW is incorporated, the process has the potential to recycle a large amount of LW. Nanni et al. [1] used an internal mixer to prepare composites of LW with polyamide 12 (PA-12), thermoplastic polyurethane (TPU), polylactic acid (PLA), and a thermoplastic elastomer (TPE). They incorporated up to 10% (*w*/*w*) LW with a 0.2 mm average particle size and concluded that the best mechanical properties were obtained with the PLA matrix, as it presents the best leather dispersion and strongest PLA–LW interactions. Also, Liu and co-authors [9] reported an improvement in tensile strength and elongation at break for poly(vinyl alcohol (PVA)–LW composites for LW incorporations of up to 15%. The improvements are ascribed to the excellent adhesion between PVA and LW, whose size was previously reduced to a micrometric granulometry of 0.068−0.35 mm, following a multi-step pan-mill milling process that also chemically activated the leather fibers.

On the other hand, Ramaraj et al. [10] studied the incorporation of up to 15% LW in acrylonitrile–butadiene–styrene (ABS) and concluded that its presence only slightly affected tensile and flexural strengths, Izod impact strength, and abrasion resistance, but tensile modulus and elongation at break were significantly reduced. Noyon et al. [11], while using linear low-density polyethylene (LLDPE) to incorporate up to 10% LW in the form of buffing dust (≤0.5 mm), reported an expressive increase in tensile strength and a decrease in elongation at break. In conclusion, the best reinforcement properties are reached when the polymer contains functional groups, such as hydroxyl or carboxylic groups, which can interact with amide or hydroxyl groups present in LW. Additionally, fine LW particles seem to be crucial for improving the dispersion of the additive within the polymer matrix.

The properties of rubber–LW composites have also been extensively studied. Ferreira and co-authors [12] studied the incorporation of LW in styrene–butadiene rubber (SBR) and nitrile–butadiene rubber (NBR) in amounts up to 300 phr, and Renivaldo et al. [13] performed the same study with SBR (up to 80 phr). While Ferreira reported a loss of tensile strength for the composites, Revinaldo reported an increase in the same property. Since both used a two-roll mill to make the mixtures, the difference may lie in the size of the added LW particles, i.e., ≤1 mm vs. ≤0.3 mm, respectively, which enabled Revinaldo to obtain a better dispersion of the particles and avoid the formation of fiber agglomerates. Hang et al. [14] studied the NBR–LW (≤0.4 mm) composites and reported an improvement in tensile and tear strengths for additions up to 50 phr of LW and a decrease for higher incorporations. This author used an internal mixer to carry out the NBR–LW mixtures. Şaşmaz et al. [15] used a Banbury mixer, an equipment often used for rubber processing, to mix SBR with LW (≤0.4 mm), up to 20 phr. This author refers to a substantial improvement in tear strength and no effects of LW addition on the tensile strength in the case of SBR but a loss of tensile strength in the case of natural rubber.

Some authors have attributed the deterioration of rubber LW composites’ mechanical properties to the acid nature of chromium-based LW. To solve this problem, El-Sabbagh [16] proposed the neutralization of LW with ammonia or sodium formate before incorporation in NBR, achieving lower melt viscosities and higher tensile strength and Young’s modulus than with untreated LW. Ravichandran [17] neutralized LW with sodium bicarbonate and reported that neutralization enables the incorporation of higher amounts of LW in rubber without affecting the vulcanization properties, while Urrego [18], also performing the same treatment, reported an increase in the rigidity, tear strength, and abrasive wear resistance of NR composites, but a decrease in tensile strength. Note that this process involves the generation of liquid residues with chromium, raising another problem. All in all, regarding rubber–LW composites, a small LW size and an efficient mixing process seem to be key factors in achieving composites with optimized mechanical properties.

However, to the authors’ knowledge, no systematic work has been published that specifically focuses on the effect of LW size on the composite’s properties. On the other hand, leather milling is a costly and energy- and time-intensive process. Therefore, it has become crucial to study the influence of LW size on the composite processing parameters and its mechanical properties to optimize the procedure and adjust it to the desired properties. Concerning the mixing equipment, most works use the roller mill to make the LW–rubber mixtures [12,13,19,20,21,22,23,24] and only a few works use alternative equipment for rubber–leather mixing, namely, a Banbury mixer [15,18,19] or an internal mixer, [14,18,25] with excellent results in terms of mechanical properties. The authors tested the fabrication of SBR–LW composites using a two-roll mill and an internal mixer at a controlled temperature and concluded that more homogeneous mixtures were obtained with the internal mixer. Therefore, an internal mixer operating at 100 °C was used in this work to study the influence of LW size (≤0.5 to ≤3 mm) and content (up to 50 phr) on the mechanical properties of SBR–LW composites.

## 2. Materials and Methods

### 2.1. Materials

The SBR (type 1502) was first formulated with all additives, except sulfur, according to a recipe used by a local footwear company, and called SBR_F_. A local tannery supplied the leather as wet-blue production scraps (LW).

### 2.2. LW Scrap Size Reduction

The leather scraps were first dried at 80 °C for 12 h. Next, they were ground to a ≤4 mm size in an Erdwich mill. Further milling was performed in a Retsch cutting mill equipped with stainless steel plate sieve inserts with 0.5, 1, 2, or 3 mm of diameter perforations.

### 2.3. Chromium Determination in the LW

The method of acid digestion with aqua regia was followed in accordance with ISO11466:1995 [26] Two samples with 1 g of leather were weighed in an analytical balance and placed in Kjeldahl tubes, onto which 10 mL of aqua regia solution in a ratio of 1:3 *v*/*v* of nitric acid to hydrochloric acid was added. The leather dissolution was carried out with the tubes in a water bath at 90 °C for 3 h. The solutions were then filtered into a 100 mL volumetric flask and brought to the reference mark with distilled water. Finally, chromium was determined with a UNICAM 969 atomic absorption spectrophotometer (Cambridge, UK).

### 2.4. Thermogravimetric Analysis

Thermogravimetric analysis (TGA) was performed using an equipment STA 449 F5 (Netzsch, Selb, Germany). An alumina crucible was used, and the samples (about 10 mg) were heated from 20 °C to 800° at a 10 °C/min heating rate under air atmosphere.

### 2.5. Preparation of the Composites

The SBR_F_ was cut in small pieces (≤10 mm) and dried at 70 °C for 12 h before mixing with LW, which was also dried at 70 °C for 12 h. The SBR_F_–LW formulations were prepared in an internal mixer HAAKE PolyLab QC/RheoMix600 (ThermoFisher Scientific, Waltham, MA, USA) internal mixer equipped with Banbury rotors. First, the SBR was introduced in the mixture chamber and processed for 2 min for homogenization (50 rpm, 100 °C). Then, the LW was introduced stepwise into the chamber (2 min), and the mixing process proceeded for 8 min more. The compositions of the prepared mixtures are reported in Table 1. Finally, sulfur (2 phr) was mixed with the SBR–LW in a two-roll mill. The final mixtures were placed in a 20 × 12 × 0.2 (length × width × thickness) cm mold and vulcanized at 160 °C for 6 min with an applied pressure of 50 KPa. A small portion of each sample was used to study the vulcanization kinetics, which allowed us to select the vulcanization time.

From this study, several important parameters can be calculated: the time to complete 90% of the vulcanization process, t_90_; the time during which the mixture can be processed, called scorch time, t_s2_; and the Cure Rate Index (CRI), which measures the vulcanization rate (see Equation (1)) [15].

(1) $$CRI=\frac{100}{t_{90}-t_{s2}}$$

### 2.6. Mechanical Tests

The composite plates were left for one week at room temperature to complete the vulcanization, after which they were cut for the mechanical tests. Tensile tests were performed with three specimens according to EN ISO 22654 [27], with the shape of standardized dumbbell samples (length of 115 mm; thickness of 2 mm), and tested with a 100 mm/min strain rate. Abrasion was tested with three specimens, according to ISO 20871 [28]. Briefly, a cylindrical sample was mounted on the test machine and pressed against an abrasive cylindrical drum surface with an applied pressure of 10 N. The drum was rotated at 40 rpm until 84 revolutions were completed. Abrasion was calculated from the mass difference between the samples before and after the test. For the tear strength tests, three 100 mm × 15 mm specimens were used, which were cut to a depth of 40 mm. Then, the samples were fixed from the cut parts and pulled with a 100 mm/min strain rate, following the ISO 20872 [29] standard. These standards are followed for rubber testing in the footwear industry. Hardness tests were performed with the acquisition of five measurements, following ISO 868-2003 [30], by using a type A shore durometer.

### 2.7. Microscopy Analysis

Composite samples were first fractured through tensile tests. Then, the samples were coated with an Au/Pd thin film for 2 min using the SPI Module Sputter Coater equipment. The cross-section of the samples was assessed by scanning electron microscopy (SEM) using a Quanta 400 FEG ESEM/EDAX Genesis X4M system (Ametek Inc., Berwyn, IL, USA) with energy-dispersive X-ray spectroscopy (EDS).

## 3. Results and Discussion

### 3.1. LW Characterization

**Figure 1:**
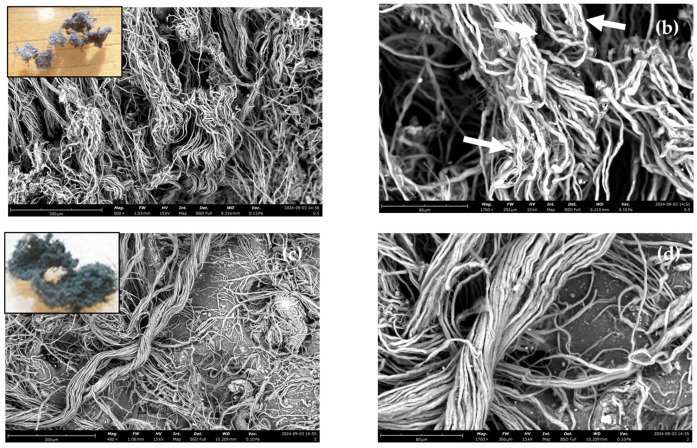
SEM images of LW ground to ≤0.5 (**a**,**b**) and ≤2 mm (**c**,**d**). The arrows indicate the regions of the fibers that present degradation.

The measured chromium content in LW was 1.7%, which is in line with the chromium contents reported in other works [12,18]. Exemplary SEM images of LW ground to ≤0.5 and ≤2 mm are shown in Figure 1a,b and Figure 1c,d, respectively. The reduction in the leather scraps to fibers was achieved in both cases, as demonstrated from the macroscopic photos of the grinded LW (see insets of Figure 1a,c). However, the fibers appear more individualized for the ≤0.5 mm LW size, with some bundles being visible for the ≤2 mm LW size (Figure 1c). Some defibrillation and fiber rupture are apparent for the ≤0.5 mm LW (see arrows in Figure 1b).

### 3.2. Rheological Properties of the Mixtures

**Figure 2:**
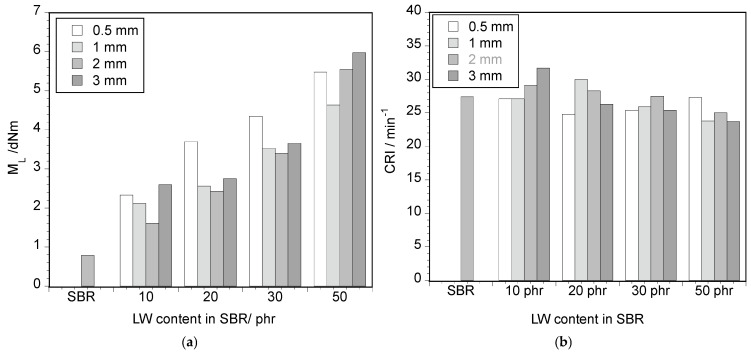
(**a**) Influence of both the size and LW content in the initial torque of the mixture (T = 160 °C). (**b**) Influence of LW content and size in the cure rate index (CRI) (T = 160 °C).

The initial torque value of the LW–SBR mixture, M_L_, is considered a measure of the viscosity of the unvulcanized sample, an important parameter by which to assess the fibers’ ability to interact with SBR and the energy consumption of the mixing process. Figure 2a shows the influence of LW size and content on the M_L_ value. The viscosity increases significantly with LW incorporation, about 200% for the 20 phr composite with particle sizes in the range ≤1 to ≤3 mm. The increase in M_L_ with LW content has been reported by several authors [15,19,21,31], and it is explained by the higher stiffness and entanglement of LW fibers and their interaction with SBR chains, bringing an additional resistance to the melt displacement. LW particle size does not significantly affect the torque value, except for the finest particles (≤0.5 mm size), for which there is an important increase in the torque value (350% in the 20 phr mixture) compared with the other sizes. This may be due to the higher interfacial area between LW and SBR, causing stronger interactions between the two materials and a higher resistance to melt movement. For the 50 phr LW content, the high amount of LW seems to determine the torque, and no differences are perceived between LW sizes.

The CRI for the mixtures, plotted in Figure 2b, indicates that the vulcanization kinetics slightly decrease with the amount of LW (about 10% for 50 phr LW, average value), which can be attributed to the obstruction of the reaction points of the SBR chains by leather fibers, thus slowing down the vulcanization process. Also, the dilution of the vulcanization agents, due to the presence of leather, should contribute to the decrease in the vulcanization kinetics. Finally, Natchimuthu [23] states that the acidity of leather neutralizes the leather curatives, also slowing down the curing process. This trend is corroborated by some authors [13,23], while others report an increase in the vulcanization kinetics [14,15] ascribed to the presence of reactive functional groups in leather fibers, which act as activators and accelerate the reaction rate.

It is possible that in the case of this work, the high amount of silica that was added to the rubber composition (~30% in weight) already played the role of an obstruction agent, partially masking the other effects and contributing to a smooth decrease in CRI.

### 3.3. Mechanical Properties

Fibers are often incorporated into a polymer matrix to reinforce the mechanical properties of the polymer. Indeed, fibers are usually mechanically stronger than polymers, with higher tensile strengths and elastic moduli. Therefore, if a strong interfacial bond exists between the two materials to enable an efficient polymer-to-fiber stress transfer, the composite is expected to see its properties reinforced, namely, tensile strength and elastic modulus, leading to a stiffer but stronger material [32]. On the other hand, a weak or non-existent polymer-to-fiber affinity degrades the mechanical properties of the matrix because not only is the stress transfer effect not possible, but voids will form at the interface between the materials [19].

**Figure 3:**
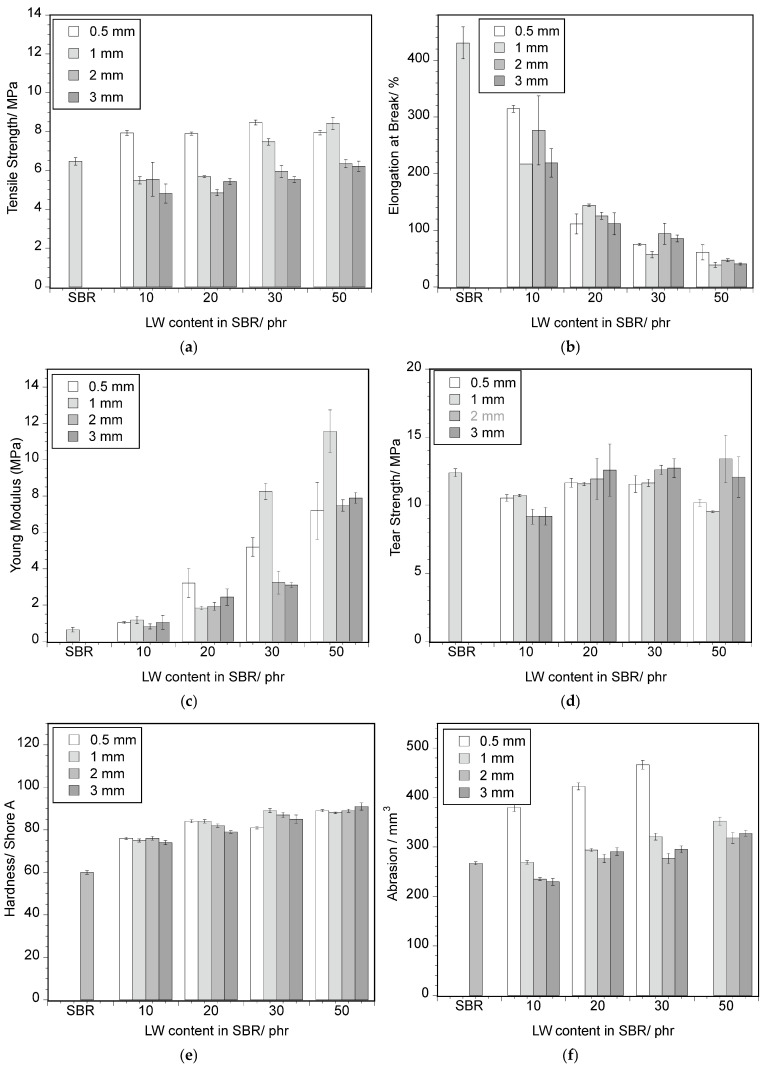
Mechanical properties of SBR–LW composites as a function of LW content and size: (**a**) tensile strength; (**b**) elongation at break; (**c**) Young’s modulus; (**d**) tear strength; (**e**) hardness; (**f**) abrasion.

The graph describing the relationship between tensile strength and LW content and size is reported in Figure 3a. A decrease in tensile strength is identified for low LW contents, except for the finer LW size (≤0.5 mm). However, this loss of tensile strength is not as expressive as the ones verified in other works [18,19]. On the contrary, for the ≤0.5 mm composites and for the 30 phr and 50 phr (LW with ≤1 mm), the tensile strength is higher than for SBR. This is an indication that for the finer granulometries, there is a more efficient stress transfer from SBR to the fibers, possibly because of the higher interfacial SBR–fibers contact area and lower fiber entanglement than in the case of the coarser sizes (see Figure 1). The improvement of mechanical properties for the finer LW particles is in good agreement with other works [14,21,31].

The elongation at break decreases, and the Young’s modulus increases with the increase in LW incorporation for all the LW sizes (~−70% and ~240%, respectively, for the 20 phr composite), as seen in Figure 3b,c. This is because leather fibers display an elongation at break of less than 1% [33] and a higher Young’s modulus than SBR. Furthermore, SBR chains lose mobility due to entanglement and interaction with leather fibers, which are stiffer and less mobile, resulting in a stiffer and less elastic material [34]. This effect is more pronounced for the higher LW contents (30 and 50 phr) and finer LW sizes. No significant influence of the LW size on the elongation at break was found in the tested range, but stiffness seems to increase with the incorporation of lower particle sizes.

Many authors have reported an increase in tear strength with LW incorporation, which is explained by the ability of the fibers to distribute the load and bridge the formed cracks, thereby increasing the fracture energy [12,33]. However, in this study, such an effect was not observed, as shown in Figure 3d, where the tear strength was not significantly affected by the LW content or the size of the added leather particles. The hardness and abrasion of the SBR composites, as a function of the LW size and content, are shown in Figure 3e,f, respectively. The hardness of the composites increases with the increase in LW content (45%, 20 phr of LW) due to the lower mobility and stiffness of the LW fibers and their negative effect on the SBR chain’s ability to respond to stress [12,21]. No influence of LW particle size is perceived. The abrasion, in turn, smoothly increases with the increase in LW content (~10%, 20 phr LW), except for the ≤0.5 mm LW addition, whose behavior sets it apart from that of other particle sizes. Abrasion is much higher in this case (~60%, 20 phr LW), which may be associated with a higher degree of fiber individualization that facilitates mechanical removal of the material. The general increase in wear with LW incorporation is in good agreement with other studies [19,21].

### 3.4. Microstructural Analysis

**Figure 4:**
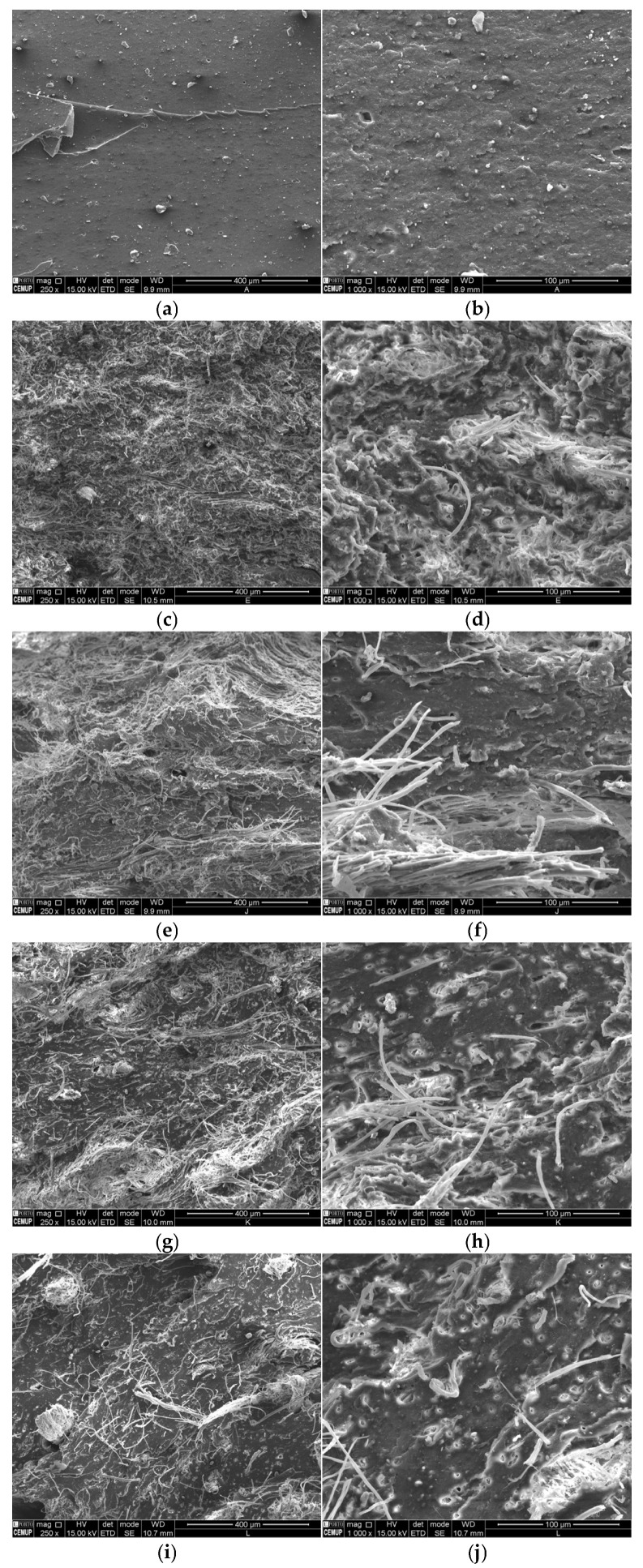
SEM micrographs of SBR (**a**,**b**) and SBR–LW composites with 20 phr of LW with undersizes of 0.5 mm (**c**,**d**), 1 mm (**e**,**f**), 2 mm (**g**,**h**), or 3 mm (**i**,**j**).

**Figure 5:**
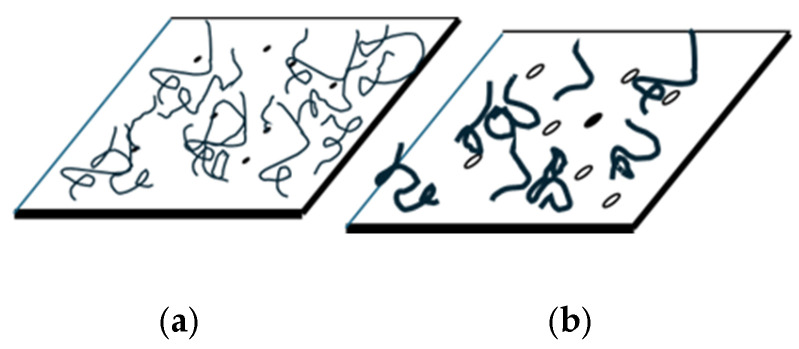
Illustration of the fracture diagrams for (**a**) finer fibers and (**b**) coarser fibers. The full circles represent the broken fibers that remained adhered to the SBR, and empty holes represent spaces that were filled by fibers that were torn off.

Exemplary cross-sections of SBR–LW composites after fracture via tensile tests are shown in Figure 4 for 20 phr of incorporated LW, with particle sizes ranging from ≤0.5 to ≤3 mm. Contrary to the smooth fracture surface displayed for SBR, the composite fracture surfaces are rough due to the existence of torn-off/fractured fibers and holes left by torn-off fibers (compare Figure 4b,d). The best LW dispersion in the SBR matrix was obtained for the ≤ 0.5 mm LW size, with the LW fibers more evenly distributed in the SBR matrix, and with fewer agglomerates than for the larger particle sizes, as shown, for example, in Figure 4c,g. We suggest that the more even and individualized fiber distribution will potentiate the SBR–to-leather stress transfer, leading to higher tensile strengths and Young’s moduli, as observed in Figure 3a,c, and lower elongation at break (Figure 3b). Furthermore, the number of holes due to fibers tearing off is lower in the case of the ≤0.5 mm size, suggesting that the fracture mechanism of the composites may change depending on the LW size: fiber breaking for the finer sizes, and fiber tearing off in the case of the coarser sizes, as illustrated by Figure 4d,j and Figure 5 for a conceptual diagram.

The reasons for this behavior are twofold. First, a stronger interfacial contact should exist in the case of the lower LW sizes because these fibers were chemically activated by the high grinding shear stress, which translated into breakage and defibrillation (Figure 1b). The fiber activation during the grinding process was reported by Liu et al. [9] to justify the strong adhesion to the polyvinyl alcohol matrix. Second, a higher surface area exists for the finer fiber granulometries due to disentanglement and defibrillation processes. Therefore, for the same force, the stress on the fiber–SBR interface will be lower, contributing to a better adhesion resistance.

### 3.5. Thermal Properties

**Figure 6:**
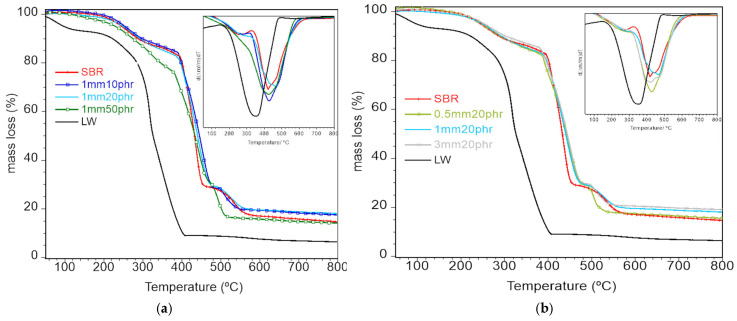
Thermogravimetric curves for the SBR–LW composites as a function of LW content (**a**) and size (**b**). The insets show the derivatives of the curves.

The thermogravimetric analyses of the SBR–WL composites are reported in Figure 6, together with the thermogravimetric curves for SBR and LW. In Figure 6a, the LW curve shows a first mass loss of 7% for temperatures up to 120 °C, due to the evaporation of humidity, and a second mass loss of 86% in the 200–400 °C temperature range, ascribed to collagen decomposition [10]. The 7% residual mass is associated with inorganic compounds that were added for hide treatment and tanning, e.g., chromium salts.

For the SBR–LW composites, the first mass loss, up to ~100 °C, is related with the evaporation of residual water adsorbed to the LW. It is important to note that a ~1% mass decrease is observed for all composites, except for the 50 phr one (~3%), due to the higher amount of LW. The main mass loss occurs between ~150 and 450 °C (~70%) for all composites and is due to the degradation of both the collagen and rubber chains, as can be concluded from the curves for LW and SBR. A final degradation stage (500–550 °C) is observed, and in the case of natural rubber, it was ascribed to the decomposition of long rubber chains [31]. The 15–18% residual mass at high temperatures is due to the presence of LW inorganic additives and silica and zinc oxide from SBR.

By comparing the composites thermogravimetric curves, it is possible to conclude that the thermal resistances are similar to that of SBR, except for the 50 phr composite, where the TG curve is shifted to lower temperatures. This is more clearly visible in the derivatives graph (inset of Figure 6a) and is related to the high amount of LW. Prochon [21] and Shabani [20] performed similar studies and concluded that leather does not influence the thermal stability of the rubber–leather composites, but they used a lower leather content of 10% and 5%, respectively. Figure 6b shows that there is no influence of LW size on the thermal resistance of the composites. To acquire an insight into the first degradation stage of the composites, the temperatures at which the composites display a 5% mass loss were measured (T^5%^) and are reported in Table 2.

A slight increase in T^5%^ is observed for low amounts of LW, indicating an increase in thermal stability, which may be related to the increase in rubber cross-linking in the presence of leather [12]. However, for high amounts of LW, the lower thermal stability of LW prevails and the thermal resistance decreases. No significant influence of the LW size on T^5%^ was detected.

## 4. Conclusions

In this study, chrome-tanned LW was incorporated into SBR in the range of 0−50 phr for leather undersizes of 0.5, 1, 2, and 3 mm. The importance of this topic is twofold: to clarify the importance of leather size in the physicochemical and processing properties of the composites, and to optimize the time and production costs of LW–SBR composites, depending on the application.

It was concluded that LW is more evenly dispersed in the SBR matrix for the ≤0.5 mm size, with more individualized fibers and even some fiber defibrillation. A better fiber dispersion would probably be obtained for the other LW sizes with higher rotor speeds. During processing, the presence of LW leads to an increase in the melt viscosity, especially for the ≤0.5 mm LW. The vulcanization kinetics are little affected by either LW content or size.

The tensile strength slightly decreases with LW incorporation (~15%), except for the ≤0.5 mm LW composite, where an increase of 20% was observed (20 phr composites). A substantial increase in the Young’s modulus and a decrease in elongation at break were observed with LW incorporation, translating to composites stiffer than SBR. Furthermore, the hardness increased with the LW content, and the wear rate was shown to be particularly high for the ≤0.5 mm LW size. The thermal stability of the composites decreases for high amounts of LW, but no influence of LW size was noticed.

To conclude, the SBR–LW composites can be used in applications where limited elongations at break of about 120% is acceptable. If the tensile strength cannot be lower than that of SBR, LW with particle sizes no higher than 0.5 mm must be added to the mixture. Tear strength and hardness are not affected by the LW particle size in the range of ≤0.5 mm to ≤3 mm, but fine LW particles should be avoided if the material is to be used in wear conditions.

## Figures and Tables

**Table 1:** Composition of the mixtures used in the composites.

Reference	Particles Size (mm)	LW Content (phr)			
SBR_F_		0	0	0	0
Comp10_0.5	≤0.5	10	0	0	0
Comp20_0.5		20	0	0	0
Comp30_0.5		30	0	0	0
Comp50_0.5		50	0	0	0
Comp10_1.0	≤1.0	0	10	0	0
Comp20_1.0		0	20	0	0
Comp30_1.0		0	30	0	0
Comp50_1.0		0	50	0	0
Comp10_2.0	≤2.0	0	0	10	0
Comp20_2.0		0	0	20	0
Comp30_2.0		0	0	30	0
Comp50_2.0		0	0	50	0
Comp10_3.0	≤3.0	0	0	0	10
Comp20_3.0		0	0	0	20
Comp30_3.0		0	0	0	30
Comp50_3.0		0	0	0	50

**Table 2:** Temperatures for a 5% mass loss of the composites.

LW content (phr)	SBR	10	20	50
T^5%^ (°C)	247	254	250	230
LW size (mm)	SBR	0.5	1	3
T^5%^ (°C)	247	248	248	252

## Data Availability

The authors confirm that the data supporting the findings of this study are available within the article.
